# Dansyl-Labelled Ag@SiO_2_ Core-Shell Nanostructures—Synthesis, Characterization, and Metal-Enhanced Fluorescence

**DOI:** 10.3390/ma13225168

**Published:** 2020-11-16

**Authors:** Elżbieta Szczepańska, Anna Synak, Piotr Bojarski, Paweł Niedziałkowski, Anna Wcisło, Tadeusz Ossowski, Beata Grobelna

**Affiliations:** 1 Faculty of Chemistry, University of Gdansk, Wita Stwosza 63, 80-308 Gdańsk, Poland; elzbieta.szczepanska@phdstud.ug.edu.pl (E.S.); pawel.niedzialkowski@ug.edu.pl (P.N.); anna.wcislo@ug.edu.pl (A.W.); tadeusz.ossowski@ug.edu.pl (T.O.); 2 Faculty of Mathematics, Physics and Informatics, University of Gdansk, Wita Stwosza 57, 80-308 Gdańsk, Poland; piotr.bojarski@ug.edu.pl

**Keywords:** dansyl-labelled Ag@SiO_2_ core-shell nanostructures, plasmonic platform, enhanced fluorescence, fluorescence decay

## Abstract

The present work describes synthesis, characterization, and use of a new dansyl-labelled Ag@SiO_2_ nanocomposite as an element of a new plasmonic platform to enhance the fluorescence intensity. Keeping in mind that typical surface plasmon resonance (SPR) characteristics of silver nanoparticles coincide well enough with the absorption of dansyl molecules, we used them to build the core of the nanocomposite. Moreover, we utilized 10 nm amino-functionalized silica shell as a separator between silver nanoparticles and the dansyl dye to prevent the dye-to-metal energy transfer. The dansyl group was incorporated into Ag@SiO_2_ core-shell nanostructures by the reaction of aminopropyltrimethoxysilane with dansyl chloride and we characterized the new dansyl-labelled Ag@SiO_2_ nanocomposite using transmission electron microscopy (TEM) and Fourier-transform infrared spectroscopy (FTIR). Additionally, water wettability measurements (WWM) were carried out to assess the hydrophobicity and hydrophilicity of the studied surface. We found that the nanocomposite deposited on a semitransparent silver mirror strongly increased the fluorescence intensity of dansyl dye (about 87-fold) compared with the control sample on the glass, proving that the system is a perfect candidate for a sensitive plasmonic platform.

## 1. Introduction

In the last few decades, core-shell type nanostructures have been of interest due to their unique properties. These structures belong to the biphasic materials consisting of an inner core structure and an outer shell, which both can either be an organic polymer [1,2] or an inorganic compound such as: TiO_2_ or SiO_2_ [3]. The core-shell nanostructures have been invented due to the extraordinary properties of the shell material, which can improve the reactivity, thermal stability, or oxidative stability of the core structure. The combination of two or more types of materials with different properties into one particle in the form of a core-shell structure is an effective way to obtain a multifunctional material. For instance, magnetic core-shell nanostructures functionalized by ethylenediaminetetraacetic acid (EDTA) were used as a novel magnetic adsorbent for Cd^2+^ and Pb^2+^ ions binding in an aqueous medium [4], as well as the determination of Cd^2+^, Pb^2+^, and Cu^2+^ by amino modified Fe_3_O_4_@SiO_2_ nanoparticles [5].

Recently, core-shell nanostructures have been selected as excellent candidates for applications in various branches of nanotechnology, including biosensing [6], targeted therapies [7], and metal enhanced fluorescence (MEF) [8]. The inorganic-organic nanocomposites of core-shell types that absorb in an ultraviolet/visible range and emit in a visible spectral domain can be particularly useful for bio-imaging [9], sensors design [10], safe sun-screens [11], and modern, very efficient, and selective drug carriers [12].

Metal enhanced fluorescence is a consequence of the electromagnetic coupling between the transition moments of fluorophores and surface plasmons induced by the optical excitation. Surface plasmons are coherent and collective electron oscillations confined at the dielectric–metal interface. The surface plasmons can accumulate the optical field and energy on the nanoscale and enhance various light–matter interactions [13]. The emergence of such a wave may be caused by the absorption of electrons from a beam of appropriate energy falling on the surface of a thin metallic layer or photons ranging from ultraviolet to near infrared by various types of metallic nanostructures. The enhancement effect depends on several factors, e.g., the thickness of the dielectric layer, distribution of the distance between the fluorophores and the metallic surface, plasmon resonance conditions, and the type of metal (the so-called SPR-ratio) [14,15]. Fluorescence is stronger in the presence of small silver nanoparticles deposited on the substrate surface, due to two simultaneously occurring effects—local electric field enhancement and radiative decay engineering (RDE). In the first effect, the interaction of the impinging light with small noble metal particles creates localized plasmons, which locally can significantly enhance the electric field. This increases the rate of excitation. The second effect is an interaction of the excited fluorophore with the nanoparticles. This interaction leads to a rapid release of the excitation energy and its radiation to the free space. This effect results in an increase of a quantum yield of the molecule and in the decrease of its lifetime. Such a combination is possible only with the increasing radiative constant rate. Therefore, this effect is also known as radiative decay engineering [16,17,18,19,20,21,22,23].

In the absence of the metal, the quantum yield and lifetime are given by:

Q_0_ = Γ/Γ_0_+k_nr_ (1)

τ_0_ = 1/Γ+k_nr_ (2)

where Γ is the radiative rate and k_nr_ is the nonradiative decay rate.

In the presence of the metal, the quantum yield and lifetime are presented by Equations (3) and (4), respectively:

Q_m_ = Γ+Γ_m_/Γ+ Γ_m_+k_nr_+k_m_ (3)

τ_m_ = 1/Γ+Γ_m_+k_nr_+k_m._ (4)

The effects of local field enhancement and RDE, contribute to the total fluorescence enhancement. Due to this phenomenon, fluorophores can emit significantly more intensive light in well-defined conditions and remain photostable for a longer period of time. This is particularly important in the case of light sensitive bio-species, weakly fluorescent fluorophores, species at a very low concentration, or species distributed exclusively in the local nanostructures present in the bulk material [22,24,25].

The core-shell nanostructures labelled with multiple fluorophores can also act in the nanoscale as artificial antenna-like systems. Due to the fluorophores proximity, the system can significantly enhance the light collection efficiency in selected small volumes where the core-shell structure is located. In particular, the use of a core-shell with multiple rather closely located fluorophores may also be suitable to study larger macromolecules, as it can amplify the nonradiative energy transfer making this well-established spectroscopic ruler sensitive even at distances significantly larger than 10 nm [26].

The dansyl (DNS) group was selected, as it is widely used to modify amino acids, specifically, for protein sequencing and amino acid analysis by the reaction with DNS chloride used as the reagent [27,28,29]. This group is used to study fluorescent conjugates of albumin, for protein conformational changes studies, and active site characterization. Moreover, DNS derivatives can be used for the documents protection or it can be used for a quantitative assessment of certain types of hair damage [30].

In this work, we present a novel antenna-like core-shell system of an original design. It seems that such a combination of the antenna effect and the plasmonically induced enhancement may lead to stronger fluorescence enhancements. Silver nanoparticles were selected to form the core due to their high enhancement efficiency of the fluorescence signal [31]. In addition, silver has an antimicrobial effect and already in small quantities exhibits a preservative effect in cosmetics, textile, and medical products [32,33]. Silica shell was chosen as a separator to control the distance between the DNS group used as fluorophore and the silver surface to obtain maximum fluorescence enhancement. Additionally, silica shell usually increases the chemical stability of the silver core [34].

**Scheme 1:**
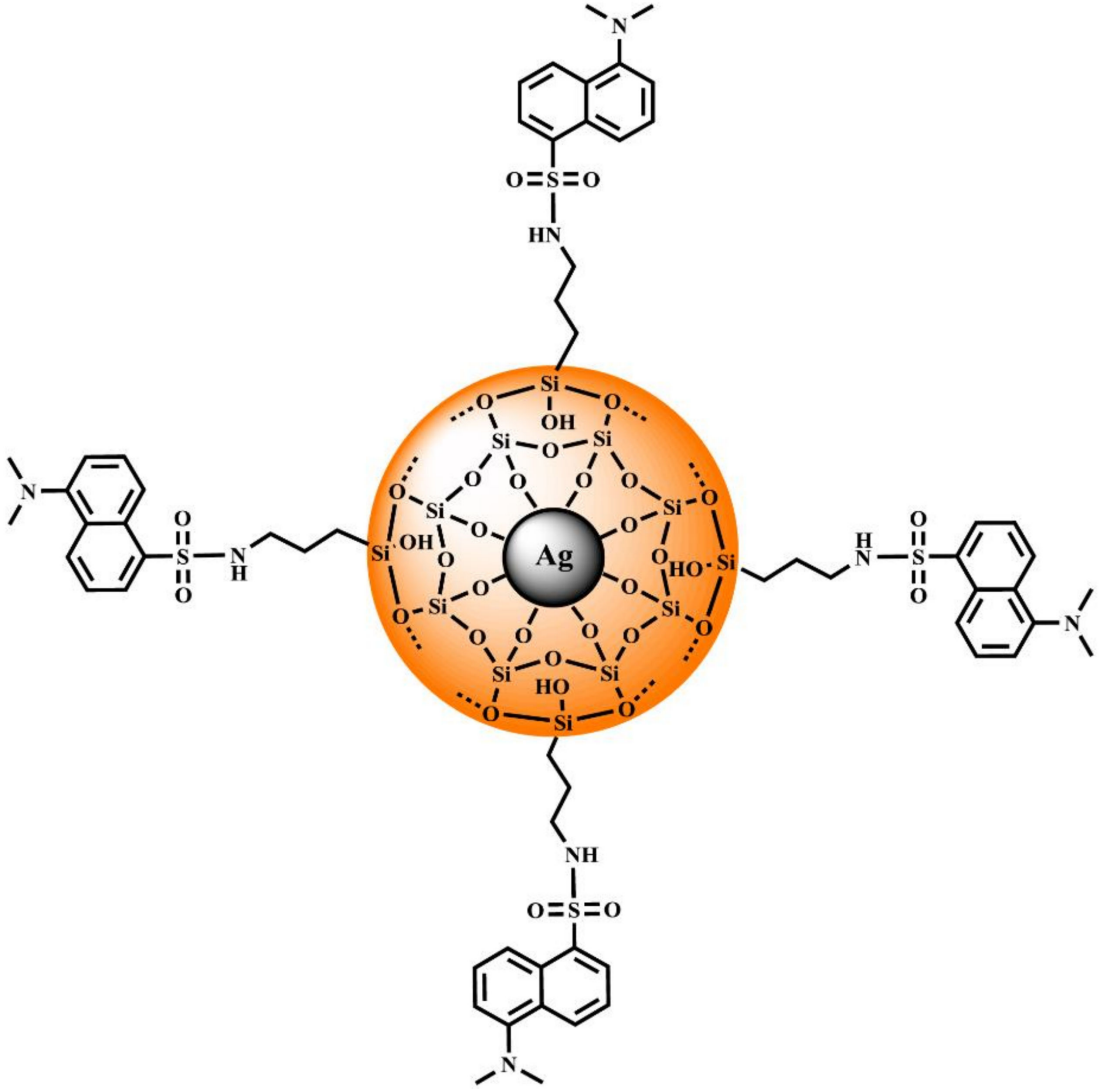
Proposed structure of Ag@SiO_2_-NH-DNS.

The main objective of this work is the synthesis and analysis of the emissive properties of the novel core-shell structure consisting of Ag@SiO_2_ with an additional modification using DNS fluorophores attached to the shell in a controllable way by the reaction of 3-(aminopropyl)trimethoxysilane with DNS chloride. The proposed structure of the designed nanocomposite is visualized in Scheme 1.

We expect that this relatively simple and specifically designed system will provide a significant enhancement of the fluorescence intensity due to the combination of plasmonic and antenna-like effect compared with the control sample—(5-(dimethylamino)-N-propylnaphthalene-1-sulfonamide (*n*-Pr-NH-DNS). Such systems are necessary to design a new generation of plasmonic platforms dedicated to biosensing or emission amplifiers in optoelectronic applications.

## 2. Materials and Methods

### 2.1. Materials and Reagents

All the reagents and solvents were used without further purification. Silver nitrate (AgNO_3_, 99.99%), sodium citrate, ammonia, tetraethyl orthosilicate (TEOS), 3-(aminopropyl)trimethoxysilane, (APTMS, 97%), toluene, diethyl ether, dichloromethane, dansyl chloride (5-(dimethylamino)naphthalene-1-sulfonyl chloride, DNS-Cl), triethanolamine, *n*-propylamine, methylene chloride, triethylamine were purchased from Sigma-Aldrich (Poznan, Poland). All the samples were prepared using deionized water. Silver mirrors were purchased from Thin Metals Films Ltd. (Basingstoke, UK).

### 2.2. Synthesis

#### 2.2.1. Ag@SiO_2_-NH_2_

In the first stage of Ag@SiO_2_-NH_2_ synthesis, the Ag@SiO_2_ was obtained according to the method described by Synak et al. [8]. Then, 0.7 mL of APTMS was added to 0.3 g of Ag@SiO_2_ suspended in 3 mL of toluene in a bottom flask equipped with a reflux condenser. The reaction mixture was kept at 120 °C for 24 h. After cooling down to room temperature, the crude product was separated by the centrifugation at 5000 rpm for 5 min and the obtained white precipitate was washed twice with diethyl ether and methylene chloride to remove the excess of APTMS and was finally dried in a nitrogen stream.

#### 2.2.2. Ag@SiO_2_-NH-DNS

A total of 539 mg of dansyl chloride (2.00 mmol) dissolved in 10 mL of toluene was added dropwise to a suspension of 442 mg of Ag@SiO_2_-NH_2_ and 150 μL of triethylamine in 40 mL of toluene. The reaction mixture was kept at 80 °C for 24 h. After disappearance of the mixture yellow color, the desired product was separated by the centrifugation at 5000 rpm for 5 min, washed with methylene chloride to remove the excess of dansyl chloride, and was finally dried in a nitrogen stream.

#### 2.2.3. n-Propyl Dansyl Amide (n-Pr-NH-DNS)

A total of 500 mg of dansyl chloride (1.85 mmol) dissolved in 10 mL of toluene was added dropwise to a mixture of *n*-propylamine (1.85 mmol, 155 µL) and triethylamine (200 µL) in 40 mL of toluene. The reacting mixture was kept in 50 °C for 24 h. The progress reaction was monitored using TLC plates (silica gel 60, Merck, 0.25 mm). After cooling to room temperature, the solvent was evaporated under the reduced pressure and the obtained crude product was dissolved in 100 mL of methylene chloride and washed with water (2 × 100 mL). The organic layer was removed under the reduced pressure giving 501 mg of the desired product with a 91% yield.

TLC (SiO_2_), (dichloromethane); Rf = 0.22.

MALDI—TOF MS: m/z 293.14 [M+H]^+^, (MW = 292.12).

^1^H-NMR (CDCl_3_, 500 MHz): δ (ppm): 0.65–0.68 (t, 3 H, CH_3_-CH_2_-CH_2_-,J_1_ = J_2_ = 7.5 Hz); 1.275–1.347 (sxt, 2 H, CH_3_-CH_2_-CH_2_-,J_1_ = 7.0 Hz, J_1_ = 7.5 Hz, J_2_ = J_3_ = J_4_ = J_5_ = 7.25 Hz); 2.747–2.775 (t, 2 H, CH_3_-CH_2_-CH_2_-, J_1_ = J_2_ = 7.0 Hz); 2.791 (s, 6 H, CH_3_-N); 4.880–4.904 (t, 1 H, CH_3_-CH_2_-CH_2_-NH, J_1_ = J_2_ = 6.0 Hz); 7.079–7.094 (d 1 H, H-6 Ar, J_1_ = 7.5 Hz); 7.406–7.438 (t, 1 H, H-7 Ar, J_1_ = J_2_ = 8 Hz); 7.440–7.473 (t, 1 H, H-3 Ar, J_1_ = 8.5 Hz, J_2_ = 8.0 Hz J_2_ = 8.25 Hz); 8.154–8.170 (dd 1 H, H-8 Ar J_1_ = 1.0 Hz J_1_ = 7.0 Hz); 8.239–8.257 (d 1 H, H-2 Ar J_1_ = 9.0 Hz); 8.438–8.455 (d 1 H, H-4 Ar J_1_ = 8.5 Hz).

^13^C-NMR (CDCl_3_, 125 MHz): δ (ppm): 11.03, 22.95, 45.08, 45.41, 115.17, 118.82, 123.18, 128.35, 129.52, 129.68, 129.91, 130.33, 134.92, 151.3.

### 2.3. Methods

Transmission electron microscopy (TEM) measurements were performed with Tecnai G2 Spirit BioTWIN by FEI (Eindhoven, the Netherlands). Samples were dispersed in ethanol and sonicated.

FT-IR spectra were obtained with a Bruker IR IFS66 Fourier transform infrared spectrometer (Ettlingen, Germany). Samples were prepared by the standard KBr pellet method.

^1^H-NMR spectra were recorded with a Bruker AVANCE III 500 MHz spectrometer in room temperature using deuterated chloroform as the solvent. The chemical shifts (δ) were given in ppm relative to tetramethylsilane (TMS) used as the internal standard, coupling constants (J) were given in (Hz). The following abbreviations were used to describe multiplicities: (s) Singlet, (d) doublet, (dd) doublet of doublets, (t) triplet, (sxt) sextet.

Matrix-assisted laser desorption/ionization–time-of-flight mass spectrometry (MALDI-TOF/MS) was performed using a Bruker Autoflex maX MALDI TOF spectrometer (Billerica, MA, USA).

The fluorescence spectra and fluorescence intensity decays were measured upon front face excitation at λ_exc_ = 370 nm with the laser head LDH-D-C-370 controlled with PDL 800-D, PCI-board for TCSPC TimeHarp 200 (Pico-Quant, Germany) [35]. Fluorescence light was recorded by the H10721P-01 photomultiplier (Hamamatsu Photonics K.K., Japan) using the slit of Czerny-Turner spectrograph Shamrock 303i-B (Andor Technology, UK). Fluorescence intensity decays were analyzed using the FluoFit Pro software (PicoQuant, Germany).

The contact angle technique involves the measurement of the contact angles of a drop of water on the surface of a tested sample. At first, the structure was dissolved in ethanol, dispersed by ultrasound, spotted onto a glass slide, and allowed to dry. A 2 μL drop of water was deposited using a syringe onto a sample placed on a glass slide. The image of the drop was recorded with a CCD camera, and after digital image analysis the average contact angle was determined by the Young-Laplace method from the angles measured on both sides of the drop. Measurements were repeated 30 times. Wettability measurements were carried out using a Krüss DSA100 goniometer (Hamburg, Germany).

## 3. Results and Discussion

### 3.1. Synthesis and Characterization of Nanocomposite

**Scheme 2:**
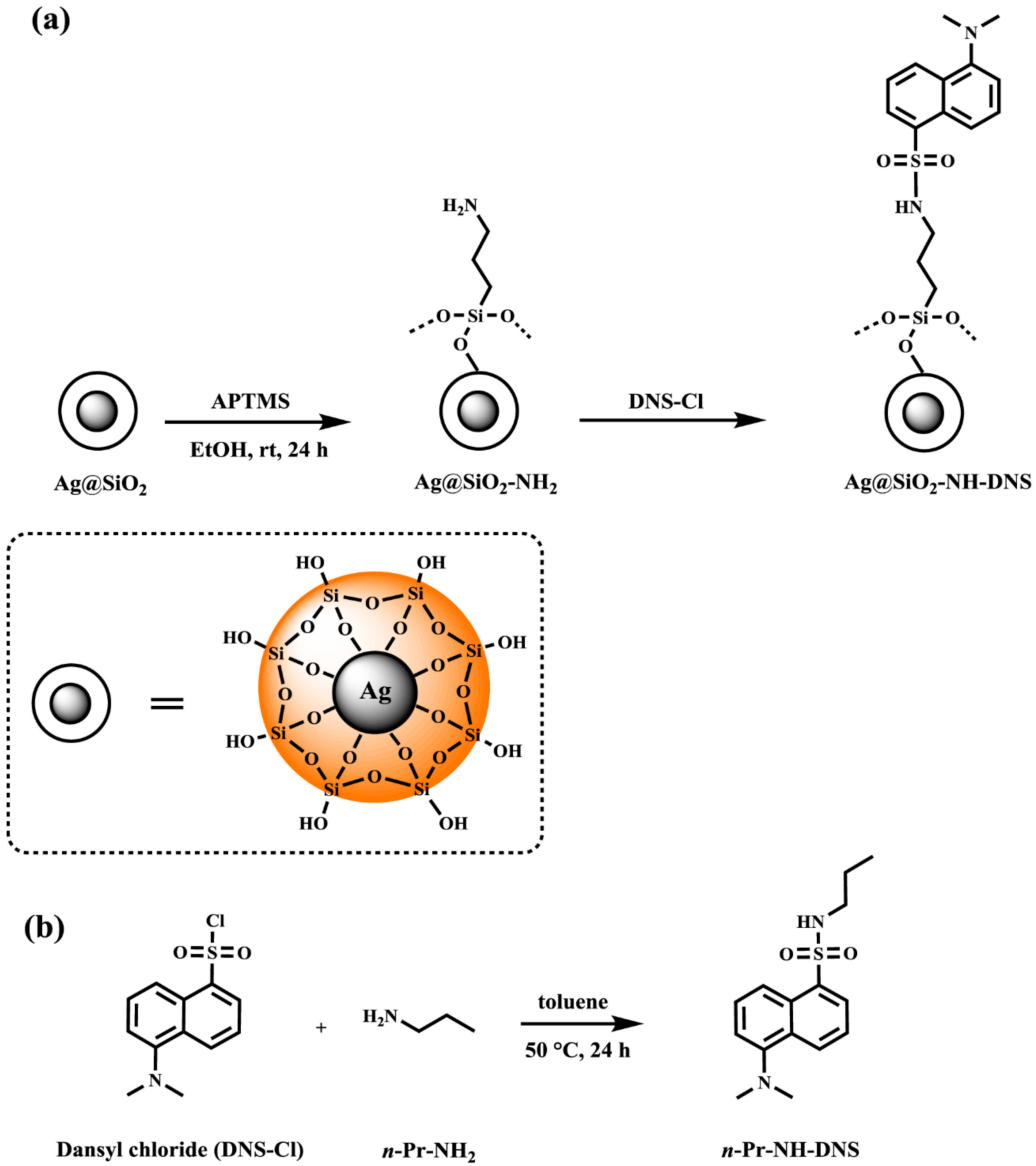
The synthesis route to (**a**) Ag@SiO_2_-NH-DNS and (**b**) *n*-Pr-NH-DNS.

As illustrated in Scheme 2a, the preparation of this nanocomposite was achieved in several steps. During the first step, the silver nanoparticles were prepared via the reduction of silver nitrate in an aqueous solution in the presence of trisodium citrate according to the procedure described previously [8]. Then, the obtained nanoparticles were coated with a silica nanolayer in the reaction of tetraethyl orthosilicate to obtain a spherical core-shell using the Stöber method [36]. This process consists of hydrolysis and then condensation of tetraethyl orthosilicate occurring in the alkaline conditions by the addition of ammonia to the ethanol solution [37]. The amino-functionalized Ag@SiO_2_ nanoparticles were prepared according to the modified procedure described previously to obtain the core-shell magnetic nanomaterial [38,39]. These nanoparticles were obtained in the reaction of silica coated nanoparticles with APTMS in refluxing toluene (24 h duration time). The amino-functionalized core-shell silver nanomaterial was used for further modification based on the reaction with DNS chloride conducted in toluene in the presence of triethylamine at 80 °C for 24 h. The reaction was performed until all the used substrates in the reaction mixture were consumed (Scheme 2a). The number of the attached groups on the core-shell surface was about 6 to 9 µmol/g, which was calculated based on the method used in peptide chemistry [40]. Moreover, we determined the concentration of DNS present in Ag@SiO_2_-NH-DNS (2 × 10^−5^ M).

The synthesis of *n*-Pr-NH-DNS used as a control sample was performed using DNS chloride and *n*-propylamine as substrates (Scheme 2b). Elevation of the reaction temperature and utilization of toluene as the reaction solvent significantly increased the reaction efficiency. The yield of performed reaction is comparable to those reported in the literature and exceeds 90% [41,42].

### 3.2. Transmission Electron Microscopy—Morphology Analysis

The silver nanoparticles can be applied as the fluorescence enhancers due to their surface plasmon resonance in the visible spectral range. Therefore, keeping in mind the spectroscopic characteristics of Dansyl dye, these particles were used also in this work to build the nanocomposite core [22].

**Figure 1:**
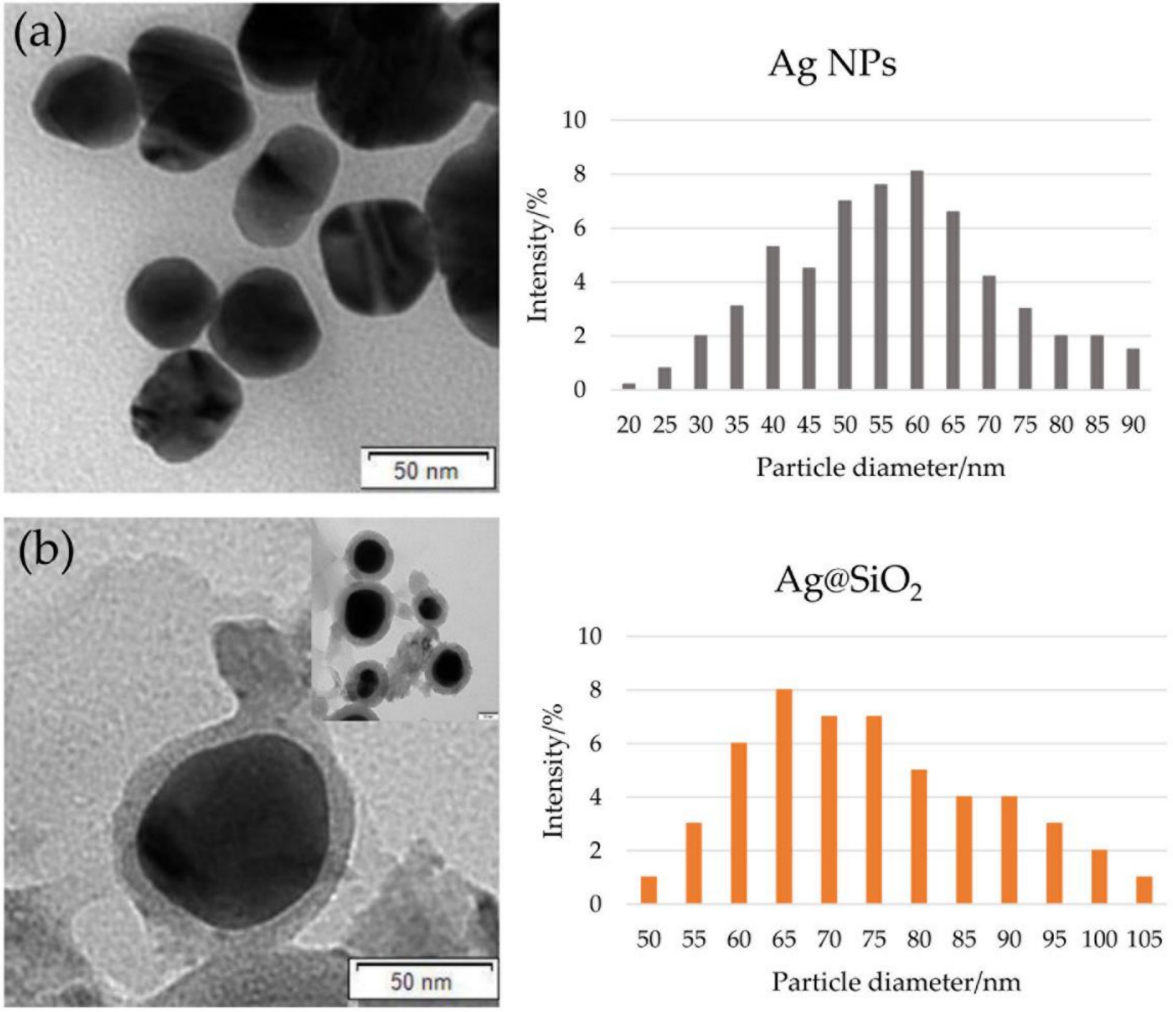
TEM image (left panels) and diagrams of the particle diameter (right panels) obtained for (**a**) Ag NPs and (**b**) Ag@SiO_2_.

In order to determine the morphology of the designed material the transmission electron microscopy (TEM) was used. The silver nanoparticles image is shown in Figure 1a (left panel), the size of the statistical representation of the silver nanoparticles obtained in the synthesis was between 20 and 90 nm and as seen in Figure 1a (right panel). According to our experiences, these parameters are optimal to obtain high fluorescence enhancement [22].

Figure 1b (left panel) shows a typical micrograph of the studied core-shell nanocomposite. Particles of quite a broad diameter distribution can be observed. This can be seen in the histogram (Figure 1b, right panel). The diameter of the statistical representation of core-shell nanostructure presented in Figure 1 is about 70 nm, with the thickness of silica shell about 10 nm.

The conditions for the synthesis of core-shell nanostructures were optimized to enable the shell preparation with the defined size and to obtain the maximum fluorescence enhancement. The maximum fluorescence enhancement is usually obtained with the optimal fluorophore-surface distance of about 10–15 nm [43]. If the fluorophore is located closer than 4 nm to the metal surface, a strong fluorescence quenching and a lifetime shortening is observed as a result of the nonradiative energy transfer to the metal core. At distances exceeding 15–20 nm, the fluorescence enhancement progressively decreases due to the gradual decoupling of the fluorophore-plasmon pair [44].

### 3.3. FT-IR Spectroscopy Analysis

**Figure 2:**
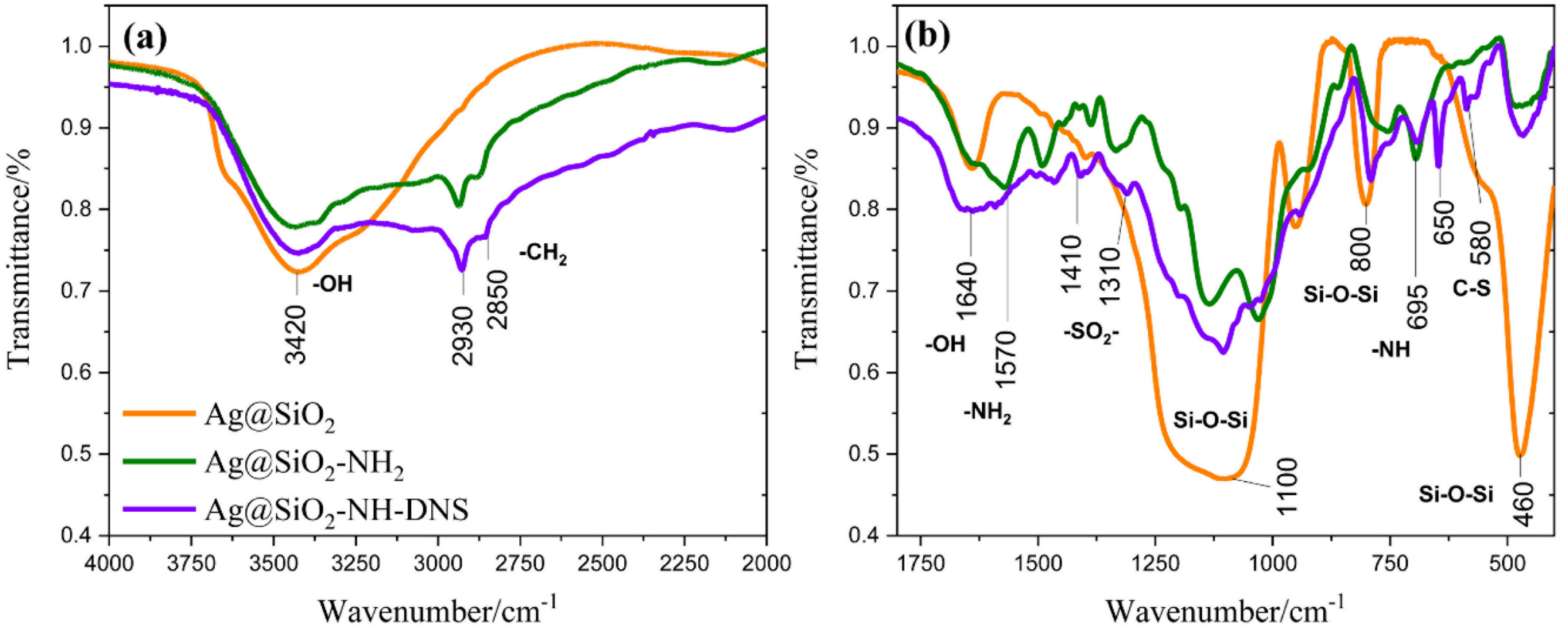
FT-IR spectra in range (**a**) 4000 to 2000 cm^−1^ and (**b**) 1800 to 400 cm^−1^ for Ag@SiO_2_, Ag@SiO_2_-NH_2_, and Ag@SiO_2_-NH-DNS.

Here, we decided to carry out the FT-IR measurements of the obtained materials to identify the functional groups. This technique provides information on the backbone structure of both the siloxane network, as well as the organic groups present at the surface of the material [45]. Figure 2 shows the FT-IR spectra for: Ag@SiO_2_, Ag@SiO_2_-NH_2_, and Ag@SiO_2_-NH-DNS, respectively. The spectrum of core-shell nanostructures (Ag@SiO_2_) exhibits several oscillation bands related to Si-O-Si vibrations. The wide and strong broad band with the maximum located at 1100 cm^−1^ is typical for asymmetric stretching vibrations of the Si–O-Si group. The absorption band at 950 cm^−1^ can be ascribed to the bending vibration of Si-OH group, but the peak at 800 cm^−1^ can be associated with the Si–O–Si symmetric stretching vibrations [46,47]. We also propose that the peak at 470 cm^−1^ corresponds to the bending of the bridging oxygen atom perpendicular to the Si-O-Si plane. In addition to the bands associated with Si-O-Si vibrations, there are bands in the spectrum that can be assigned to the bending vibrations of OH, such as this at 1640 cm^−1^ which can be caused by H_2_O deformation. Moreover, there are other bands corresponding to OH stretching vibrations which are located at 3420 cm^−1^, whereas the shoulder located near 3750 cm^−1^ can be attributed to the Si–OH group in the silica shell [48,49].

New characteristic bands appeared in the FTIR spectrum of the amino-functionalized core-shell nanostructures (Ag@SiO_2_-NH_2_). Bands in the 2800–3000 cm^−1^ region can be assigned to asymmetrical and symmetrical stretching vibrations of -CH_2_ groups attached to the silica surface. Moreover, they reflect the conformationally ordered or disordered states of the aminopropyl group participating in the hydrogen bonding with the silanol group of silica surface [50]. The bands at 1570 and 695 cm^−1^ belong to the N-H stretching band of -NH_2_ group and bending vibrations of -NH, respectively [51].

Furthermore, the FT-IR spectrum of Ag@SiO_2_-NH-DNS presents new peaks of the nanocomposite at 650 and 580 cm^−1^, which can be assigned to stretching vibrations of the C-S group. In addition, peaks at 1410 and 1310 cm^−1^ can be ascribed to the asymmetric stretching vibrations of the -SO_2_ group [52].

### 3.4. Contact Angle Measurement

**Figure 3:**
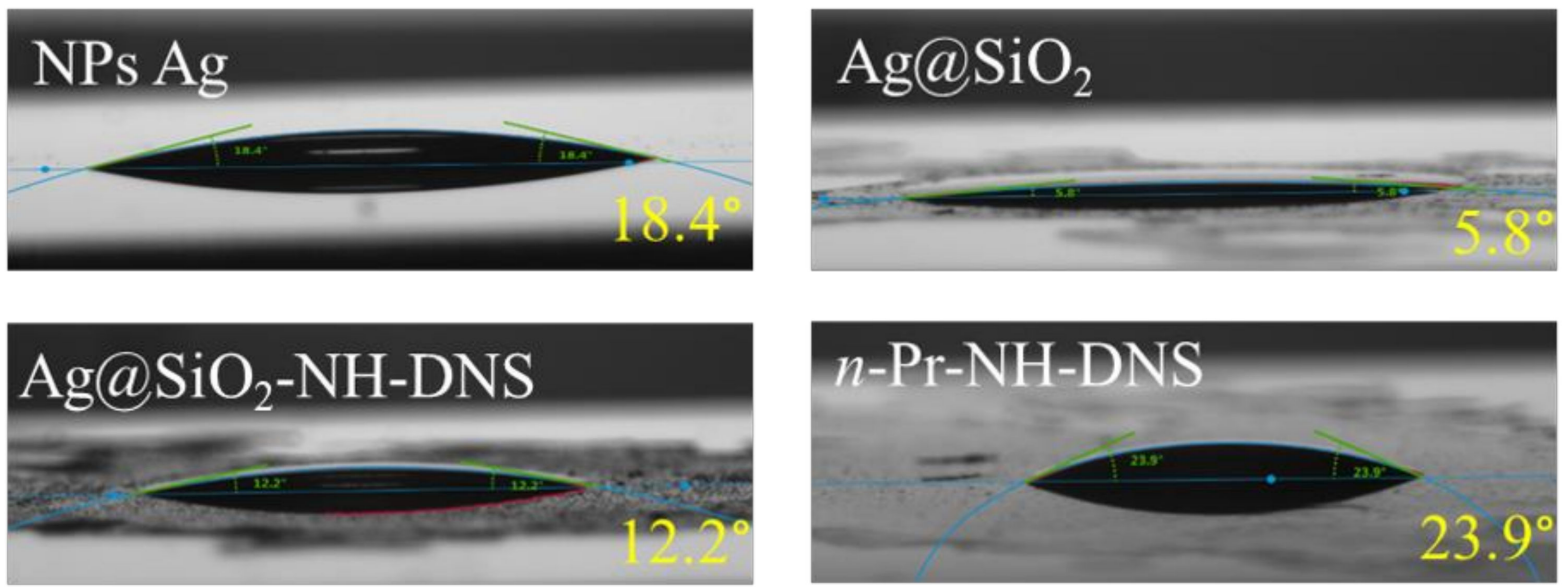
Photographs showing water contact angle measurement results for the measured samples.

Next, we conducted a series of measurements of water contact angle (WCA) for thin films composed of tested nanoparticles deposited on the glass plate surface. Water wettability measurements are used to assess the hydrophobicity and hydrophilicity of surfaces, including nanoparticles [53,54,55]. All the presented materials show good wettability, since the measured contact angles were smaller than 25° (Figure 3).

Covering the nanoparticles with a silica coating causes a significant reduction in the contact angle (from 18.4 to 5.8°). However, attachment of a DNS group to the surface of the core-shell nanoparticles leads to the increase of the hydrophobicity of such a film. The large difference (almost two-fold) between the measurement of the layer containing only DNS particles (*n*-Pr-NH-DNS), and the DNS modified core-shell film (Ag@SiO_2_-NH-DNS) results most likely from the roughness of such a layer. The layer of nanoparticles deposited on the surface of the glass plate can form a heterogeneous layer compared with the surface of the layer containing only DNS moieties. Therefore, the wettability is the result of the surface chemical nature, as well as its roughness which enhances the wettability resulting from the first factor [53].

### 3.5. Fluorescence Properties

**Figure 4:**
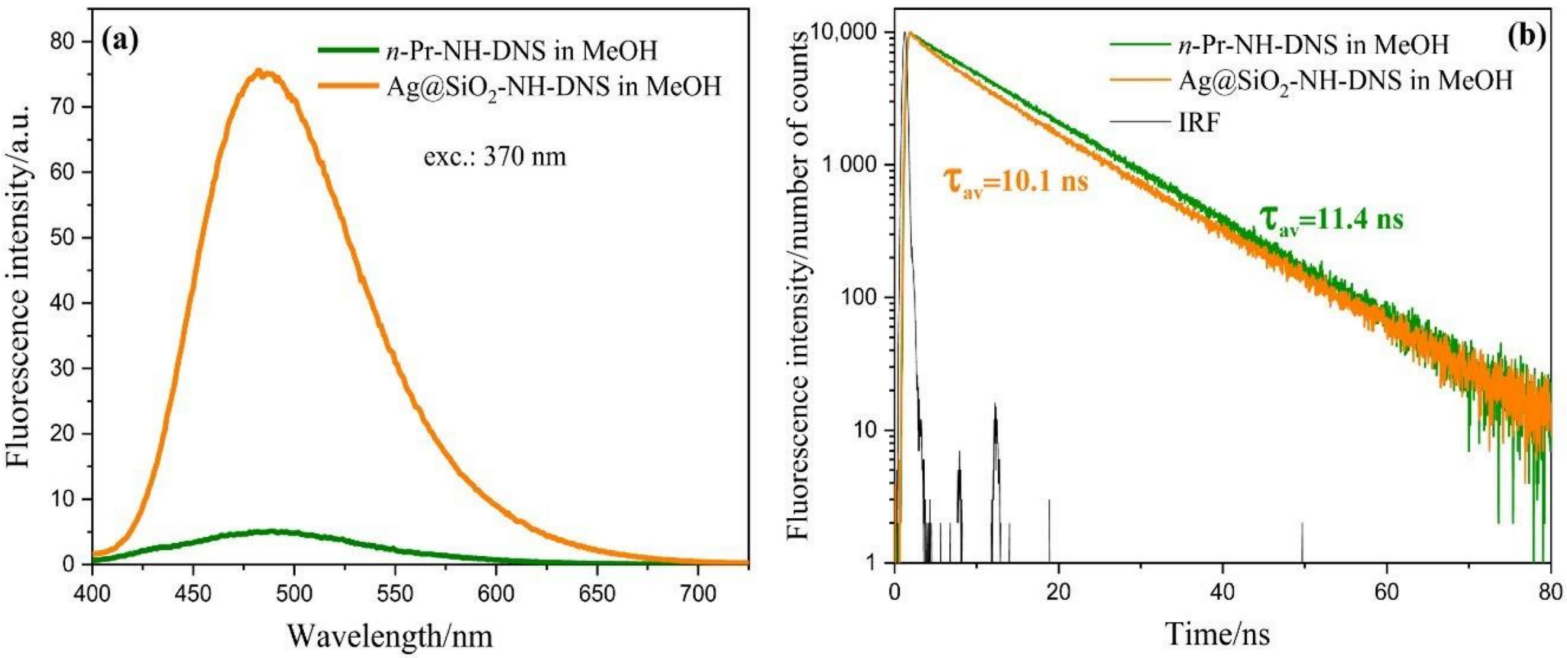
Fluorescence spectra (**a**) and fluorescence intensity decays (**b**) obtained for Ag@SiO_2_-NH-DNS and *n*-Pr-NH-DNS in the MeOH solution.

Figure 4a presents the fluorescence spectra of DNS with Ag@SiO_2_ nanostructures modified by the covalently bound DNS group (Ag@SiO_2_-NH-DNS) in the MeOH solution (orange line) and DNS (*n*-Pr-NH-DNS) in the MeOH solution (green line). The measurements for these samples were performed at the same conditions. The maximum of all the spectra is located at 485 nm. The intensity of fluorescence spectrum of Ag@SiO_2_-NH-DNS is about 14 times higher than that of *n*-Pr-NH-DNS upon identical experimental conditions. The fluorescence enhancement effect results from the electromagnetic interaction of the plasmons present on the surface of the silver core of the nanoparticles with the set of antennas—DNS probes attached to the shell in a controllable manner. Taking into account the simplicity of this system, the obtained high enhancement is a very promising result for potential applications.

Figure 4b shows the fluorescence intensity decay of n-Pr-NH-DNS in MeOH. In the case of the Ag@SiO_2_-NH-DNS solutions in MeOH the fluorescence lifetime is about 10 ns, which is rather similar to the result obtained for the control sample without core-shell nanostructures [56].

Moreover, we performed studies using various plasmonic platforms. These platforms are considered as the type of substrates composed of the silver nanoparticles deposited and arranged on the semitransparent silver mirrors. Such substrates can improve the quality of spectroscopic and microscopic tests giving a better visualization and detection of biologically important molecules and processes, even at unfavorable conditions [57,58,59,60]. As can be seen from our results, benefits of plasmonic platforms include a many-fold stronger fluorescence. In addition, these properties strongly prolonged the photostability of samples [22].

**Figure 5:**
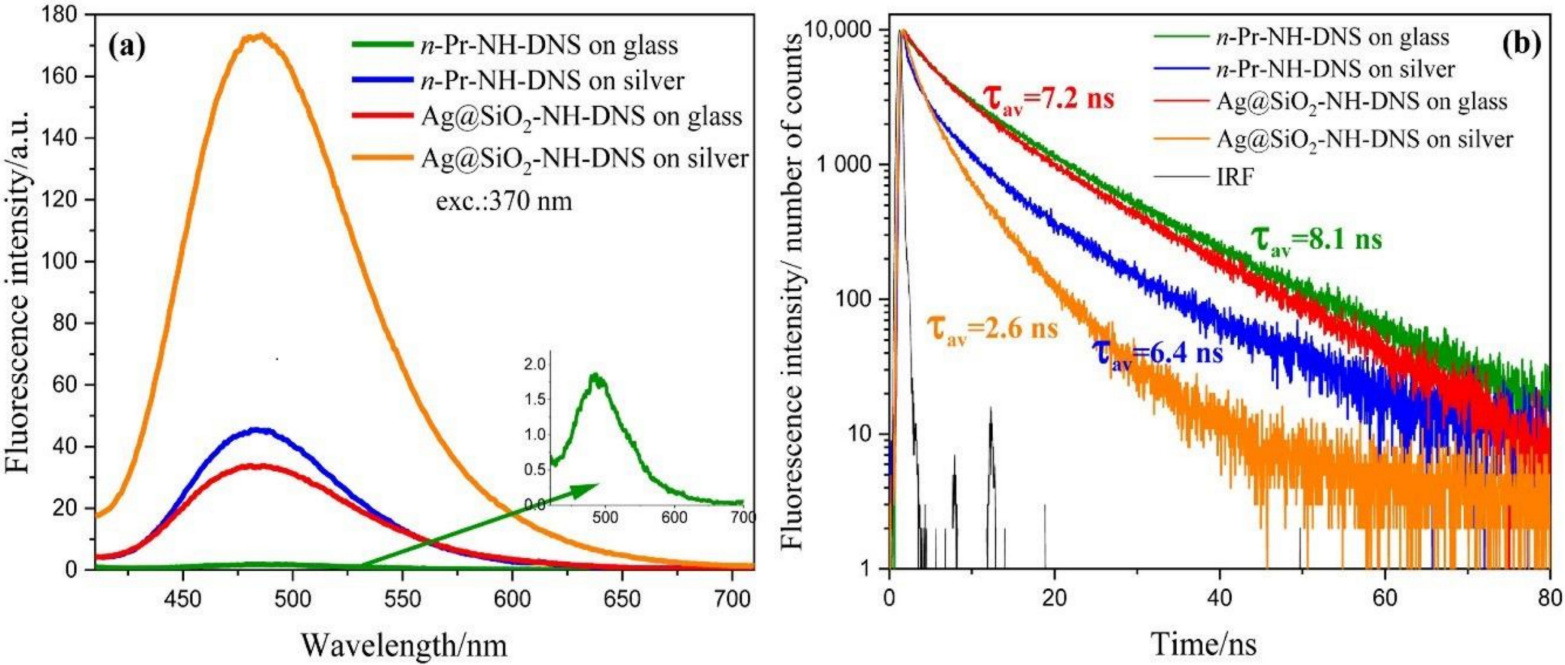
Fluorescence spectra (**a**) and fluorescence intensity decays (**b**) obtained for Ag@SiO_2_-NH-DNS and *n*-Pr-NH-DNS on plasmonic platforms and glass.

Figure 5a presents fluorescence spectra of the Ag@SiO_2_-NH-DNS on the silver mirror (orange line), on the glass (red line), and n-Pr-NH-DNS on the silver mirror (blue line) and on the glass only (green line). In this case, the solutions were applied on the surface of silver and glass in the form of a very thin layer using the spin coating method. As can be seen, the emission signal obtained on the silver mirror has been extremely enhanced, as it is 87 times stronger compared with the emission from the same layer of the same compound under the same experimental conditions but on the glass surface. Comparing the fluorescence enhancement on the same substrates before and after the application of core shell nanostructures, the fluorescence signal is 17 times higher for dansyl on the glass and 4 times higher for the silver. The fluorescence intensity of dansyl increases 23 times on the silver substrate compared to glass, while for Ag@SiO_2_-NH-DNS nanostructures, the intensity is 5 times higher.

In summary, a strong enhancement of the emission signal can be obtained even by direct coupling of DNS molecules with the core shell nanostructures. However, even a more spectacular result can be obtained using additional silver mirrors (Figure 5a).

The enhancement efficiency of nanostructures is known to be dependent on the distance between the fluorophore and nanostructures, as well as on the topological properties of the metal surface. The short fluorophore-metal distance (less than 4 nm) usually leads to an effective fluorescence quenching as a result of nonradiative excitation energy transfer to the metal [16,35,61]. During the synthesis of the plasmonic platform discussed in this work, this unfavorable factor was minimized by selection of the appropriate thickness of the: SiO_2_ shell, SiO_2_ layer on the metal surface [8], and Ag@SiO_2_ modified using DNS moieties.

Based on our previous experiences, we selected the substrate type, the appropriate thickness of the metal layer and the semiconductor separator to obtain the plasmonic platform exhibiting the highest effectiveness [8,22,62].

Figure 5b shows the fluorescence intensity decay of Ag@SiO_2_-NH-DNS and n-Pr-NH-DNS on the plasmonic platforms and the glass. Based on this picture, one can observe that for Ag@SiO_2_-NH-DNS and n-Pr-NH-DNS on silver, the fluorescence enhancement effect is accompanied with a shortening of the fluorescence lifetime.

As expected, the shortest fluorescence lifetimes were obtained for Ag@SiO_2_-NH-DNS on the silver mirror. The mean fluorescence lifetime of Ag@SiO_2_-NH-DNS in the presence of silver mirror is about 3 times shorter than that measured for n-Pr-NH-DNS on the glass. The interaction between the excited molecules and metallic core of core-shell nanostructures leads to the increase of the radiative rate constant, resulting in the increased fluorescence quantum yield and decreased fluorescence lifetime. This mechanism, called radiative decay engineering (RDE) [17], seems to play an important role in the case of Ag@SiO_2_-NH-DNS on the plasmonic platforms. Additionally, the antenna effect can vastly increase the efficiency of the metal enhanced fluorescence. A single DNS molecule is replaced by the multiple separate but closely located molecules on the shell surface. DNS molecules interact with the light-induced localized plasmons on the core-shell nanostructures and are additionally exposed to the extremely strong local electric fields generated by the interaction of localized plasmons with plasmons traveling in a conductive silver surface. The second mechanism which causes a strong enhancement of DNS emission is related to the interaction of exciting light with Ag@SiO_2_ nanostructures. This interaction creates localized plasmons which can locally enhance the electric field, therefore, increasing the excitation rate. This effect was observed for other fluorophores on the plasmonic platforms prepared using different techniques [19,23].

## 4. Conclusions

We successfully designed and tested novel core-shell optically active hybrid nanostructures (Ag@SiO_2_-NH-DNS), as an element of a new plasmonic platform, to vastly enhance the fluorescence intensity. The nanocomposite consists of Ag NPs used as an enhancer core and the amino-functionalized silica shell used as a separator between the silver nanoparticles and DNS dye covalently attached to the outer surface of silica. In comparison with the control sample—5-(dimethylamino)-N-propylnaphthalene-1-sulfonamide—the intensity of fluorescence is 14 times higher due to the electromagnetic interaction between the plasmons on the silver core surface and the transition moments of antennas—DNS probes attached specifically to the shell. Additionally, the deposition of the core-shell nanocomposites on the semitransparent silver mirrors leads to a further spectacular enhancement effect (87-fold increase) of the fluorescence intensity compared with the control sample. The obtained nanocomposite proved to be photostable and durable for a significantly longer time than necessary to perform and repeat all the measurements.

Therefore, the designed plasmon core-shell system is promising for potential use in the nanoscale optical signal enhancement, especially for biosensing purposes, which will be soon covered in our upcoming report.
